# Synergistic Co-activation Increases the Extent of Mechanical Interaction between Rat Ankle Plantar-Flexors

**DOI:** 10.3389/fphys.2016.00414

**Published:** 2016-09-21

**Authors:** Chris Tijs, Jaap H. van Dieën, Guus C. Baan, Huub Maas

**Affiliations:** ^1^Department of Human Movement Sciences, Faculty of Behavioral and Movement Sciences, MOVE Research Institute Amsterdam, Vrije Universiteit AmsterdamAmsterdam, Netherlands; ^2^Department of Organismic and Evolutionary Biology, Harvard UniversityCambridge, MA, USA

**Keywords:** soleus, gastrocnemius, plantaris, connective tissue, Achilles tendon, co-activation

## Abstract

Force transmission between rat ankle plantar-flexors has been found for physiological muscle lengths and relative positions, but only with all muscles maximally activated. The aims of this study were to assess intermuscular mechanical interactions between ankle plantar-flexors during (i) fully passive conditions, (ii) excitation of soleus (SO), (iii) excitation of lateral gastrocnemius (LG), and (iv) during co-activation of SO, and LG (SO&LG). We assessed effects of proximal lengthening of LG and plantaris (PL) muscles (i.e., simulating knee extension) on forces exerted at the distal SO tendon (F_SO_) and on the force difference between the proximal and distal LG+PL tendons (ΔF_LG+PL_) of the rat. LG+PL lengthening increased F_SO_ to a larger extent (*p* = 0.017) during LG excitation (0.0026 N/mm) than during fully passive conditions (0.0009 N/mm). Changes in F_SO_ in response to LG+PL lengthening were lower (*p* = 0.002) during SO only excitation (0.0056 N/mm) than during SO&LG excitation (0.0101 N/mm). LG+PL lengthening changed ΔF_LG+PL_ to a larger extent (*p* = 0.007) during SO excitation (0.0211 N/mm) than during fully passive conditions (0.0157 N/mm). In contrast, changes in ΔF_LG+PL_ in response to LG+PL lengthening during LG excitation (0.0331 N/mm) were similar (*p* = 0.161) to that during SO&LG excitation (0.0370 N/mm). In all conditions, changes of F_SO_ were lower than those of ΔF_LG+PL_. This indicates that muscle forces were transmitted not only between LG+PL and SO, but also between LG+PL and other surrounding structures. In addition, epimuscular myofascial force transmission between rat ankle plantar-flexors was enhanced by muscle activation. However, the magnitude of this interaction was limited.

## Introduction

Numerous animal studies have reported clear evidence that muscle force can be transmitted to the skeleton via epimuscular myofascial connections (Huijing, 2009; Maas and Sandercock, 2010). The extent of epimuscular myofascial force transmission is dependent on the position of a muscle relative to its surrounding structures (Maas et al., 2004; Meijer et al., 2006; Rijkelijkhuizen et al., 2007; Huijing and Baan, 2008). More recently, intermuscular interaction was assessed by imposing exclusively physiological muscle-tendon unit (MTU) lengths and relative positions (Bernabei et al., 2015a). Proximal lengthening of active lateral gastrocnemius (LG) and plantaris (PL) muscles (i.e., simulating knee extension) increased the force exerted at the distal tendon of the soleus (SO) muscle significantly (by 12%). This indicates that myofascial linkages can be mechanically relevant within physiological MTU lengths and relative positions.

In other studies using a more intact hindlimb and in which tendons were not disrupted from the skeleton, the mechanical relevance of intermuscular connections has been challenged (Maas and Sandercock, 2008; Tijs et al., 2015). The MTU length of bi- and poly-articular muscles was changed at their origin, by imposing changes in knee joint angle, while the MTU length of the synergistic mono-articular ankle muscles was kept constant by testing at a constant ankle angle. In those studies, the ankle moment exerted on excitation of the mono-articular muscle was not significantly affected when the length and relative position of passive synergistic muscles were changed by varying knee angle. Because the hindlimb was kept intact as much as possible, only the net moment of each muscle at the ankle joint could be assessed, and no information was available about the forces exerted at the individual tendons. Therefore, epimuscular myofascial force transmission *per se* could not be excluded.

The contradictory results of the studies described above may be explained by differences in experimental conditions. Firstly, in an intact hindlimb ankle plantar-flexion muscles merge distally into the Achilles tendon (Maas and Sandercock, 2008; Tijs et al., 2015). Although forces can be transmitted between these muscles, all force is then transmitted to the skeleton via the common Achilles tendon. As a consequence, any epimuscular myofascial force transmission may not be reflected in the moments exerted at the ankle (Tijs et al., 2015). However, similar results were found for the muscles in the anterior crural compartment, which do not share a tendon distally (Tijs et al., 2016). Secondly, the level of muscle activation and number of simultaneously excited muscles differed between the studies. While mono-articular muscles were excited maximally, synergistic muscles were either passive, partially (Maas and Sandercock, 2008; Tijs et al., 2015, 2016) or maximally (Bernabei et al., 2015a) active. As hypothesized earlier (Maas and Sandercock, 2008), muscle activation may increase the stiffness of epimuscular myofascial connections, thereby, enhancing the extent of mechanical interaction between muscles. Recently, evidence that supports this hypothesis has been provided by a study that found less shear between LG and SO fascicles in humans with LG activated than with LG passive (Finni et al., 2015), which suggests a higher stiffness of the epimuscular myofascial connections during muscle activation.

The aim of the present study was to assess effects of various combinations of SO and LG muscle activation on intermuscular mechanical interaction for physiological MTU lengths and relative positions of rat ankle plantar-flexors. Effects of LG+PL MTU length and muscle activation on i) forces exerted at the distal tendon of SO and on ii) the difference in forces exerted at the proximal and distal tendons of LG+PL were used as indications of such interaction.

## Materials and methods

### Animals

Experiments were performed on ten male Wistar rats (body mass 312.9 ± 17.7 g, mean ± s.d.). All procedures were in agreement with the guidelines and regulations concerning animal welfare and experimentation set forth by Dutch law, and approved by the Committee on Ethics of Animal Experimentation at the VU University Amsterdam (Permit Number: FBW 12-01).

According to standard procedures in our laboratory (Maas et al., 2001) the animals were deeply anesthetized by intraperitoneally injected urethane (1.2 ml/100 g body mass). If necessary, additional doses were given to suppress any reflexes. During surgery and data collection, body temperature was maintained at ~37°C. To prevent dehydration of exposed tissues, saline solution was applied regularly. At the end of the measurements, animals were euthanized with an overdose of intracardially-injected pentobarbital sodium followed by a double-sided pneumothorax.

### Surgery

The surgical procedures have been described in more detail elsewhere (Bernabei et al., 2015a) and will, therefore, be described only briefly here. The posterior crural compartment of the right hindlimb was exposed by removing the skin and biceps femoris muscle and the femur was exposed to allow attachment of a metal clamp. Medial gastrocnemius (MG) was removed fully without damaging muscle fibers of LG. This was done to prevent unphysiological strain between MG and LG as only the MTU length of LG+PL was changed (see Experimental protocol). All structures surrounding the SO, PL, LG muscle group were cut, but myofascial connections between their muscle bellies were left intact.

**Figure 1:**
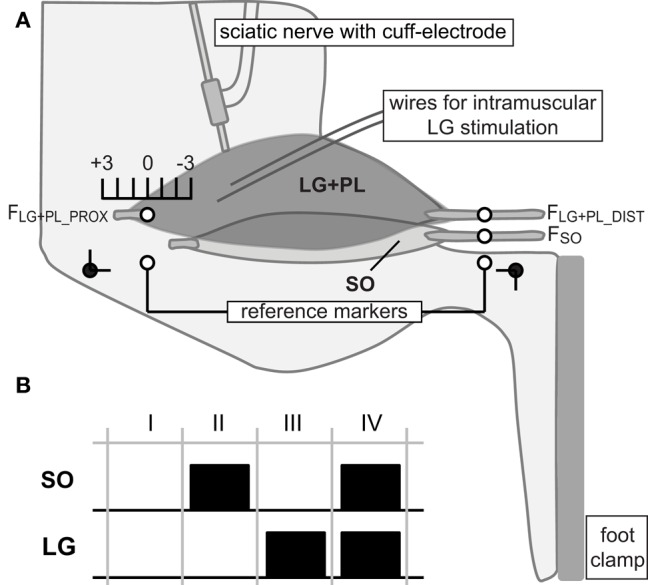
**Experimental set-up and protocol. (A)** Lateral view of the rat hindlimb in the experimental set-up with knee and ankle angle (black dots) at 90°. Proximal and distal tendons of lateral gastrocnemius and plantaris (LG+PL), as well as distal soleus (SO) tendon were connected to force transducers (F_LG+PL_PROX_, F_LG+PL_DIST_ and F_SO_, respectively). The origin of the proximal SO tendon onto the skeleton was kept intact. SO was excited via a cuff electrode around the sciatic nerve. LG was excited via intramuscular fine wire electrodes. Proximal and distal reference markers were placed on the lateral collateral ligament and distal tendon of the peroneus muscle, respectively. Markers on the distal SO tendon, distal LG tendon and proximal LG aponeurosis were placed parallel to those markers. Distal markers of SO and LG were kept at reference position. Knee extension was simulated by changing the muscle-tendon unit (MTU) length of the LG+PL complex proximally from −3 mm to +3 mm relative to the reference position (90° ankle and knee angles). **(B)** Different muscle excitation protocols used in the present study. For each LG+PL MTU length, SO and LG muscles were either passive (I), excited separately (II and III) or excited simultaneously (IV).

The hindlimb was positioned at ankle and knee angles of 90°. Markers were placed on the lateral collateral ligament and distal tendon of the peroneus muscle, and served as proximal and distal reference markers, respectively (Figure 1A). Markers on the distal SO tendon, distal LG tendon and proximal LG aponeurosis were placed parallel to the position of the reference makers. As result, changes in marker positions could be expressed relative to the reference position (L_REF_) at 90° ankle and knee angles.

LG and SO tendons merge distally into the Achilles tendon before inserting on the calcaneus. To measure individual tendon forces, these tendons were separated. The distal SO tendon was cut and connected, using Kevlar thread and a metal rod, to a force transducer (ALPHA load beam transducer, 25N maximum capacity, max output error <0.1%, compliance 0.0162 mm/N; BLH Electronics Inc., Toronto, Canada). Distal LG and PL tendons were tied together (LG+PL complex) and separated from the skeleton by cutting a small piece from the calcaneus. The proximal LG+PL tendon was separated by cutting a small bone fragment from the lateral epicondyle. Both tendons were attached to force transducers (Z6 bending beam load cell, 50N maximum capacity, max output error <0.1%, compliance 0.0048 mm/N; HBM, Darmstadt, Germany) using Kevlar thread and metal rods. The three force transducers were aligned to the muscles' lines of pull.

To excite SO, the sciatic nerve was partly dissected free for placement of a cuff electrode. In addition, all branches distally to the cuff electrode were cut, except the branch innervating SO muscle (Maas and Sandercock, 2008; Tijs et al., 2014). At the end of the experiment, it was checked whether indeed only SO muscle was excited. In five animals, a not previously reported additional branch to PL was found at the SO-PL muscle belly interface and, hence, SO was not excited exclusively. These animals were excluded from the analysis. LG has a tri-pennate structure with multiple compartments, which can be excited separately, and multiple motor endplates (Prodanov et al., 2005). Bipolar intramuscular wire electrodes were inserted near motor endplates located in the proximal region of LG. Therefore, LG was excited partially.

### Experimental protocol

SO muscle was excited by supramaximal stimulation of the sciatic nerve (amplitude: 0.4–0.5 mA, frequency: 100 Hz, pulse width: 100 μs) via the bipolar cuff electrode connected to a constant current source (Digitimer DS3, Digitimer Ltd., Hertfordshire, England). LG muscle was excited partially via the intramuscular electrodes (amplitude: 0.8–3.0 mA, frequency: 100 Hz, pulse width: 100 μs). Because only one LG compartment was stimulated, not all muscle fibers were excited. At the highest length tested (L_REF_+3 mm, see below), this yielded a force of ~3N. For the same length, but during maximal excitation of the LG+PL complex via nerve stimulation, a force of ~14 N has been found (Bernabei et al., 2015a). Because the ratio of maximal forces exerted by LG and PL is ~3:1 (Johnson et al., 2011), we estimated that 10.8 N was exerted by LG and 3.2 N by PL in the study of Bernabei et al. (2015a). Based on these data, we estimated that ~28% of LG fibers were excited in the present study.

The distal markers of SO and LG were kept at reference positions (L_REF_, see above, Figure 1A). Thus, SO was kept at a constant MTU length corresponding to a 90° ankle angle. The MTU length of the LG+PL complex was changed proximally (i.e., simulating knee extension) from −3 mm to +3 mm relative to L_REF_ with increments of 1 mm. These length changes correspond to knee joint angles of ~45° and 130°, respectively (Bernabei et al., 2015a).

For each LG+PL length, four combinations of SO and LG muscle activation were applied (Figure 1B): passive SO and LG muscles, separate excitation of either SO or LG, and simultaneous excitation of SO and LG (SO&LG).

### Data analysis

Forces exerted at the distal SO tendon (F_SO_), as well as proximal (F_LG+PL_PROX_) and distal LG+PL tendons (F_LG+PL_DIST_) were assessed before (passive force) and during SO, LG, and SO&LG excitation (total force). Total force was assessed as the mean of the total force output of the last 50 ms of tetanic stimulation.

Intermuscular mechanical interaction was assessed for different SO and LG activation protocols using two measures: (i) changes in F_SO_ with changes in LG+PL MTU length; and (ii) changes in the difference between F_LG+PL_PROX_ and F_LG+PL_DIST_ (i.e., ΔF_LG+PL_ = F_LG+PL_PROX_ - F_LG+PL_DIST_) with changes in LG+PL MTU length. ΔF_LG+PL_ was used because it is a direct indication of the net epimuscular myofascial force transmission between LG+PL and surrounding tissues (Huijing and Baan, 2001).

In addition, the time-varying force trace of F_SO_ was normalized to the mean total force for each LG+PL length during SO and SO&LG excitation. The duration of F_SO_ decrease from its peak value after the last 50 ms of tetanic stimulation to 50% of that value (half-relaxation time, HRT) was calculated. Because changes in HRT are an indication of changes in SO fascicle length (Wallinga-de Jonge et al., 1980; Maas and Sandercock, 2008), we can potentially distinguish between different mechanisms of intermuscular mechanical interaction: epimuscular myofascial connections may affect 1) SO muscle fascicle length and, therefore, the amount of force exerted by SO; or 2) the SO force distribution between intramuscular and epimuscular pathways, while the total amount of force exerted by SO is unaffected.

### Statistics

Two-way repeated measures ANOVAs (SPSS 21, IBM, USA) with “LG+PL length” and “muscle activation” as fixed factors and F_SO_ as dependent variable were performed for the combination of passive SO with either active or passive LG and for the combination of active SO with either passive or active LG. Two-way repeated measures ANOVAs with “LG+PL length” and “muscle activation” as fixed factors and ΔF_LG+PL_ as dependent variable were performed for the combination of passive LG with either active or passive SO and for the combination of active LG with either passive or active SO. In case of significant interaction effects, one-way repeated measures ANOVAs were used to test for effects of “LG+PL length” on F_SO_ and ΔF_LG+PL_ for the passive and active muscle conditions, separately. One-way repeated measures ANOVA with “LG+PL length” as fixed factor and HRT of F_SO_ as dependent variable was performed for the SO excitation and SO&LG excitation condition. Greenhouse Geisser correction was used if the assumption of sphericity was violated. Level of significance was set at *p* ≤ 0.05.

## Results

### Effects of muscle activation and LG+PL MTU length on F_SO_

**Figure 2:**
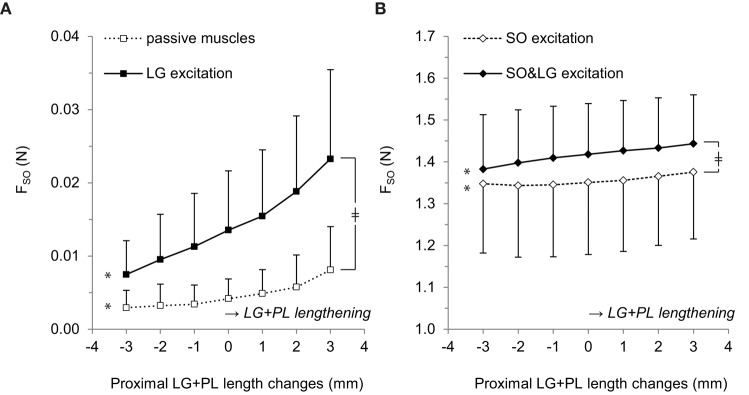
**Effects of LG+PL length changes on forces exerted at the distal tendon of SO. (A)** Distal SO tendon forces for passive muscles (□) and for active LG only (■). **(B)** Distal SO tendon forces for active SO only (♢) and for active SO and LG muscles (♦). Means ± s.d. are shown (*n* = 5). Significant effects of LG+PL length are indicated as ^*^p ≤ 0.05. Significant interaction effects between muscle excitation and LG+PL length are indicated as ^‡^*p* ≤ 0.05.

ANOVA indicated a main effect of LG excitation (*p* = 0.021), a main effect of LG+PL length (*p* = 0.009) and an interaction effect between LG excitation and LG+PL length (*p* = 0.017) on F_SO_. For passive muscles, F_SO_ increased from 0.003 ± 0.002 N at L_REF_−3 mm to 0.008 ± 0.006 N at L_REF_+3 mm (*p* = 0.039, Figure 2A, see Table 1 for mean slope values). For LG excitation only, F_SO_ increased from 0.007 ± 0.005 N at L_REF_−3 mm to 0.023 ± 0.012 N at L_REF_+3 mm (*p* = 0.009).

**Table 1:** **Extent of mechanical interaction expressed as the mean slope of F_SO_ and ΔF_LG+PL_ based on the values averaged across animals for various muscle conditions**.

	**Passive muscles**	**LG excitation**	**SO excitation**	**SO&LG excitation**
F_SO_ (N/mm)	0.0009	0.0026	0.0056	0.0101
	(±0.0006)	(±0.0014)	(±0.0025)	(±0.0032)
ΔF_LG+PL_ (N/mm)	0.0157	0.0331	0.0211	0.0370
	(±0.0055)	(±0.0246)	(±0.0073)	(±0.0208)

Means ± s.d. are shown (n = 5).

For active SO conditions (Figure 2B), ANOVA indicated a main effect of LG+PL length (*p* = 0.001) and an interaction effect between muscle excitation and LG+PL length (*p* = 0.002) on F_SO_. However, no main effect of LG excitation on F_SO_ was found (*p* = 0.053). For SO excitation only, F_SO_ ranged from 1.35 ± 0.17 N at L_REF_−2 mm to 1.38 ± 0.16 N at L_REF_+3 mm (*p* = 0.006, Figure 2B), which is a 2.4% change expressed relative to the force exerted at L_REF_. During SO&LG excitation, F_SO_ increased from 1.38 ± 0.13 N at L_REF_−3 mm to 1.44 ± 0.12 N at L_REF_+3 mm (*p* = 0.001), which is a change of 4.3% relative to the force exerted at L_REF_. These results indicate that effects of LG+PL MTU length changes on F_SO_ were enhanced by synergistic muscle activation.

**Figure 3:**
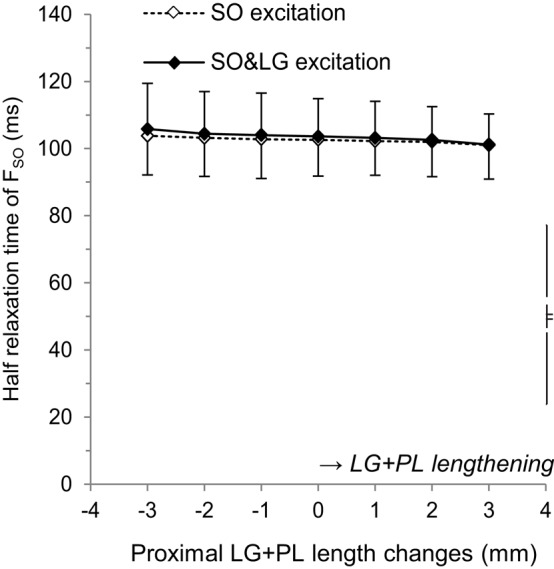
**Effects of LG+PL length changes on the half relaxation time of forces exerted at the distal tendon of SO for SO excitation (♢) and SO&LG excitation (♦)**. Means ± s.d. are shown (*n* = 5).

ANOVA indicated no effect of LG+PL length on the HRT of F_SO_ (Figure 3), both during SO excitation (average across LG+PL lengths: 102.5 ± 9.9 ms, *p* = 0.102) and during SO&LG excitation (average across LG+PL lengths: 103.5 ± 10.5 ms, *p* = 0.081). These results suggest that SO fascicle length was not affected by LG+PL length.

### Effects of muscle activation and LG+PL MTU length on ΔF_LG+PL_

**Figure 4:**
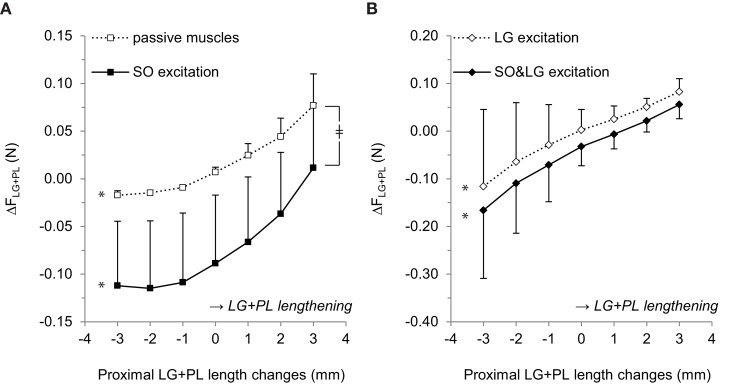
**Effects of proximal LG+PL length changes on the difference between force exerted at the proximal and distal LG+PL tendons (ΔF_LG+PL_). (A)** ΔF_LG+PL_ for passive SO and LG muscles (□) and for active SO only (■). **(B)** ΔF_LG+PL_ for active LG only (♢) and for active SO and LG (♦). Negative values of ΔF_LG+PL_ indicate F_LG+PL_PROX_ < F_LG+PL_DIST_. Means ± s.d. are shown (*n* = 5). Significant effects of LG+PL length are indicated as ^*^p ≤ 0.05. Significant interaction effects between muscle excitation and LG+PL length are indicated as ^‡^*p* ≤ 0.05.

ANOVA indicated a main effect of LG+PL length (*p* = 0.002), a main effect of SO excitation (*p* = 0.041), and an interaction effect between SO excitation and LG+PL length (*p* = 0.007) on ΔF_LG+PL_. For passive muscles, ΔF_LG+PL_ changed from −0.017 ± 0.005 N at L_REF_−3 mm to 0.077 ± 0.033 N at L_REF_+3 mm (*p* = 0.002, Figure 4A). For SO excitation only, ΔF_LG+PL_ ranged between −0.112 ± 0.068 N at L_REF_−2 mm and 0.012 ± 0.068 N at L_REF_+3 mm (*p* = 0.002). These results indicate that, when LG is passive, SO muscle activation affected the relationship between LG+PL MTU length and ΔF_LG+PL_.

For active LG (Figure 4B), ANOVA indicated a main effect of LG+PL length (*p* = 0.038) and a main effect of additional SO excitation (*p* = 0.024) on ΔF_LG+PL_, but no interaction effect (*p* = 0.161). For LG excitation only, ΔF_LG+PL_ changed from −0.116 ± 0.162 N at L_REF_−3 mm to 0.083 ± 0.028 N at L_REF_+3 mm (Figure 4B). During SO&LG excitation, ΔF_LG+PL_ changed from −0.166 ± 0.143 N at L_REF_−3 mm to 0.056 ± 0.030 N at L_REF_+3 mm.

For both passive and active LG conditions, ΔF_LG+PL_ was more negative in response to SO excitation, which implies either an increased F_LG+PL_DIST_ or reduced F_LG+PL_PROX_. Because passive forces are small at low LG+PL MTU lengths (e.g., L_REF_−3 mm: F_LG+PL_PROX_: 0.002 ± 0.001 N, F_LG+PL_DIST_: 0.019 ± 0.004 N), these results indicate that active force generated by SO muscle fibers was exerted at the distal LG+PL tendon.

## Discussion

We found mechanical interaction between ankle plantar-flexors for physiological ranges of muscle displacements, as demonstrated by changes in F_SO_ and ΔF_LG+PL_ with an increase in LG+PL MTU length. The extent of such interaction was enhanced by muscle activation, except for the effect of additional SO activation on ΔF_LG+PL_ in the condition that LG was already active. Although these results are largely in agreement with an earlier postulated hypothesis (Maas and Sandercock, 2008), the magnitudes of myofascial forces were limited.

### Differences between changes in ΔF_LG+PL_ and F_SO_

The force difference between the proximal and distal tendons of LG+PL is a direct measure of net epimuscular myofascial force transmission between LG+PL and its environment (Huijing, 2009; Maas and Sandercock, 2010). The change in F_SO_ with changes in LG+PL MTU lengths is a measure of the extent of mechanical interaction between LG+PL and SO. In the present study, we found that the mean slopes of ΔF_LG+PL_ with changes in LG+PL MTU lengths were higher than the mean slopes of F_SO_ (see Table 1). This difference in slopes can be caused by (i) force transmission from LG+PL to adjacent structures other than SO, such as the neurovascular tract supplying LG+PL, and/or (ii) changes in the amount of force transmitted between LG+PL and the proximal tendon of SO. Although these pathways could not be distinguished in the present study, the amount of force transmission via each pathway was recently estimated using a phenomenological lumped-parameter model (Bernabei et al., 2016). It was found that, for most of LG+PL MTU lengths tested, the stiffness of the neurovascular tract supplying LG+PL was higher than that of structures between LG+PL and SO. The results from that study suggest that the difference in mean slopes of F_SO_ and ΔF_LG+PL_ found in the present study is likely the consequence of force transmitted from LG+PL to adjacent structures other than SO instead of force transmission between LG+PL and the proximal tendon of SO.

### Effects of muscle (co-)activation on mechanical interaction

A previous study reported that, during maximal excitation of whole SO, LG, and PL muscles, proximal lengthening the LG+PL complex increased F_SO_ by ~10% (Bernabei et al., 2015a). In the present study, a lower increase in F_SO_ with LG co-activated was found (4.3%). This can be explained by the fact that, in the present study, PL was passive and LG was excited only partially (≈28%, see methods). Nonetheless, the effects of LG+PL length on distal SO tendon force during SO&LG excitation were more pronounced than during excitation of SO only, indicating increased intermuscular mechanical interaction. The combined results of the present and that of a previous study (Bernabei et al., 2015a) confirm that the extent of intermuscular mechanical interaction is dependent on the amount of synergistic muscle activation: higher levels of synergistic muscle activation (passive vs. partial stimulation vs. whole stimulation) resulted in increased mechanical interaction between LG+PL and SO.

We found significant interaction effects between LG+PL length and muscle activation on F_SO_ and ΔF_LG+PL_, which could be caused by increased stiffness of myofascial linkages (Maas and Sandercock, 2008). A recent study has found less relative displacement between human LG and SO fascicles during LG excitation than in passive muscle conditions (Finni et al., 2015). This may be explained by a higher stiffness of intermuscular myofascial linkages and, hence, an increase in the extent of intermuscular mechanical interaction.

Although effects of LG+PL length on F_SO_ increased when SO and LG muscles were excited, the highest changes in F_SO_ were still rather low (0.0101 N/mm, Table 1). Because the largest relative muscle displacement occurred proximally (i.e., LG+PL MTU length was increased proximally), the rather low mean slopes of F_SO_ suggests that proximally located connections at the interface between SO and LG+PL were slack or in the toe region of the stress–strain curve (Sandercock and Maas, 2009), and oriented such that they allow LG+PL muscle bellies to move proximally with minimal resistance.

### Direction of epimuscular myofascial force transmission

During both SO and SO&LG excitation, LG+PL length did not affect the half relaxation time of F_SO_, suggesting no changes in SO fascicle length. As SO muscle excitation was equal, the amount of force generated by SO muscle fibers can be assumed constant. Therefore, the increased slope of F_SO_ during SO&LG excitation compared to excitation of only SO should be explained by other mechanisms: with increasing LG+PL length proximally, (1) more LG muscle force is exerted on the distal SO tendon; (2) more force produced by SO muscle fibers is exerted on its own distal tendon instead of on the distal LG+PL tendon. We found that ΔF_LG+PL_ was more negative during SO excitation than during passive muscle conditions and more negative during SO&LG excitation than during LG excitation (Figure 4). A negative ΔF_LG+PL_ means that F_LG+PL_DIST_ is higher than F_LG+PL_PROX_, most likely resulting from active fore generated by SO transmitted to F_LG+PL_DIST_ (see Results). The first mechanism cannot fully explain the increased slope of F_SO_ during SO&LG excitation, because the additional forces measured at the distal SO tendon during excitation of only LG (Figure 2A) were limited. Therefore, the second mechanism is the most probable explanation of the increased interaction during SO&LG excitation. These results also indicate that the net orientation of connective tissue linkages was such that force transmission occurred mainly from SO to LG+PL and not vice versa.

### Functional relevance

As pointed out earlier (Herbert et al., 2008), maximal excitation of SO without excitation of its synergists is rarely seen during normal movements, which puts into question the physiological relevance of such an excitation protocol. Although selective activation of ankle plantar-flexion muscles has been found during prolonged, low-level static plantar-flexion in humans (Tamaki et al., 2011) and during paw-shakes in cats (Smith et al., 1980), these muscles are indeed co-activated during most activities. Co-activation of SO, MG and LG has been reported during human gait (Ishikawa et al., 2005), cycling (Wakeling, 2009) and (sub)maximal isotonic, isometric and isokinetic heel rising tasks (Ball and Scurr, 2015), as well as in rat (Roy et al., 1991; Bernabei et al., 2015b) and cat (Maas et al., 2009; Markin et al., 2012) locomotion. More specifically, near-maximal SO activation combined with submaximal MG activation has been found in upslope walking in rats (Roy et al., 1991) and cats (Pierotti et al., 1989). This indicates that the relative co-activation levels of synergistic muscles as applied in the present study are found during normal movements.

In the present study, we found epimuscular myofascial force transmission between SO and LG+PL. In an intact hindlimb, the distal tendons of LG and SO merge into the Achilles tendon (Maas and Sandercock, 2008; Tijs et al., 2015), which can potentially affect LG and SO muscle functioning (Tian et al., 2012; Tijs et al., 2014). In contrast, shear strain between triceps surae muscles (Bojsen-Møller et al., 2004; Finni et al., 2015) and non-uniform deformations within the Achilles tendon (Franz et al., 2015) have been found in humans. The latter results suggest that LG and SO transmit their forces independently to the calcaneus. Regardless the level of independence within the Achilles tendon, SO and LG do have very similar line of actions. Therefore, the results of the present study suggest that the absence of effects of knee angle (i.e., proximal lengthening GA and PL) on the ankle moment exerted by SO in cats (Maas and Sandercock, 2008) and rats (Tijs et al., 2015) may be a consequence of the similar line of action of SO and LG: proximal LG+PL lengthening minimally affects SO force output, and, although force is transmitted between these muscles, both muscles transmit force to the calcaneus along a similar line of action.

In contrast to the triceps surae, tibialis anterior (TA) and extensor digitorum longus (EDL) muscles within the anterior crural compartment do not have similar lines of action (TA: ankle inversion, EDL: ankle eversion). Even though connections between TA and EDL muscles are capable of transmitting force (Huijing and Baan, 2003, 2008; Maas et al., 2004), net effects of epimuscular myofascial force transmission on the ankle moment exerted by TA and EDL were found to be very limited (Tijs et al., 2016). This implies that these muscles act independently. Because of their opposite mechanical effects at the ankle joint, this may be advantageous for accurate control of the ankle joint. The studies on the triceps surae described above (Maas and Sandercock, 2008; Tijs et al., 2015) suggest that the mechanical relevance of epimuscular myofascial connections at the ankle joint is limited. Therefore, the presence of epimuscular myofascial force transmission found in the present study implies that epimuscular myofascial force transmission may be relevant for other physiological functions. It has been suggested that linkages between triceps surae muscles may distribute stresses and strains over multiple muscles and tendons, which might reduce local stresses (Bojsen-Møller et al., 2010). Additionally, intermuscular force transmission may result in local length changes within a length-restrained muscle. Muscle spindles within these length-restrained muscles may detect such deformations, thereby affecting sensory encoding (Smilde et al., 2016).

## Conclusions

We conclude that mechanical interaction is present between rat ankle plantar-flexion muscles for physiological muscle lengths and relative positions, and that linkages between LG+PL and SO are not the only pathway of epimuscular myofascial force transmission within this muscle group. Although the extent of intermuscular mechanical interaction is dependent on the level of muscle activation, the magnitude of force transmission is limited.

## Author contributions

CT and GB performed the experiments. CT performed the data-analysis and wrote the draft article. CT, JD, and HM contributed to conception and design of the experiments as well as interpretation of the data and revising the article. CT, GB, JD, and HM approved the published version and agree to be accountable for the content of the work.

## Funding

Supported by the Division for Earth and Life Sciences of the Netherlands Organization for Scientific Research [864-10-011].

### Conflict of interest statement

The authors declare that the research was conducted in the absence of any commercial or financial relationships that could be construed as a potential conflict of interest.
